# The complex transmission seasonality of hand, foot, and mouth disease and its driving factors

**DOI:** 10.1186/s12879-019-4153-6

**Published:** 2019-06-13

**Authors:** Jijun Zhao, Xiangyu Hu

**Affiliations:** 1 Institute of Complexity Science, Qingdao University, Qingdao, 266071 China; 2 The Department of Ecology and Evolutionary Biology, University of Arizona, Tucson, AZ 85721 USA

**Keywords:** Transmission rate, Population flux, Hand foot and mouth disease, Chinese spring festival

## Abstract

**Background:**

The transmission rate seasonality is an important index for transmission dynamics in many childhood infections, and has been widely studied in industrialized countries. However, it has been neglected in the study of pathogens in China.

**Methods:**

To understand the transmission dynamics of hand, foot and mouth disease (HFMD), we examined the transmission rate seasonality of HFMD in three provinces, Henan, Anhui and Chongqing, in China, using a dynamical stochastic SIR model. We investigated potential driving factors, including school terms, the Chinese Spring Festival period, meteorological factors and population flux for their effects on the HFMD transmission seasonality using multiple regression models.

**Results:**

The transmission rate of HFMD had complex seasonality with one large major peak in March and one small peak in autumn. School terms, the Chinese Spring Festival period, population flux and meteorological factors had combined effects on the HFMD transmission seasonality in mainland China. The school terms reflects the seasonal contact rate in Children, while the population flux and the Chinese Spring Festival period reflect the seasonal contact rate in population. They drove HFMD transmission rate seasonality in different time periods of the year in China. Contact rate seasonality in population dominated effects on HFMD transmission in February and March. The dramatic increase in transmission rate during February coincides with the Chinese Spring Festival period and high population flux in this month. The contact rate seasonality in children dominated effects on the transmission in the other months of the year in Chongqing. Meteorological factors can not solely explain the seasonality in HFMD transmission in mainland China; however, they may have combined effects with school terms and the highway passenger traffic on the transmission rate in Anhui during the fall semester.

**Conclusion:**

The transmission rate of HFMD in three provinces in China had complex seasonality. The Chinese Spring Festival period, population flux and (or) school terms explained the majority of the transmission rate seasonality of HFMD, and they drove HFMD transmission rate seasonality in different time periods of the year. The Chinese Spring Festival period dominantly caused the dramatic increase of the HFMD transmission rate during February.

**Electronic supplementary material:**

The online version of this article (10.1186/s12879-019-4153-6) contains supplementary material, which is available to authorized users.

## Background

Hand, foot and mouth disease (HFMD) is a childhood infectious disease primarily caused by enterovirus 71 (EV71) and coxsackievirus A16 (CA16). Since the first outbreak in 1957, these viruses have circulated throughout Asia and Pacific regions, including mainland China, Hong Kong, Taiwan, Japan, Thailand, Vietnam, Malaysia and Singapore [1–8]. In China, an average of about two million HFMD cases were reported every year since the establishment of the national enhanced surveillance system in 2008 [1, 9]. HFMD has the highest yearly reported incidence among childhood infections in China, and is one of the leading causes of death in childhood infections in the country [10, 11]. In December 2015 and January 2016, the Food and Drug Administration of China approved two EV71 inactivated vaccines to market [12], and some provinces including Beijing and Hunan have practiced voluntary vaccination since March 2016. However, a total of 2,468,174 cases and 220 deaths of HFMD were still reported in China in 2016 [11].

Reported cases or incidence has been analyzed to understand the epidemiology of HFMD, including epidemiology and reports of outbreaks [13, 14], risk factors [15], cyclical patterns of HFMD cases [1, 7, 9], spatiotemporal distribution of cases [3, 5], relationship of incidence cyclic pattern with meteorological factors [2, 4, 9], determinants of incidence spatial pattern [1, 16], and vaccination strategies and efforts [17, 18]. Reported cases are the statistical measure of the frequency of infections, however, it does not tell us the transmissibility of a disease. The basic reproduction number *R*_0_ and the transmission rate *β*, which can be estimated by fitting a mathematical or stochastic model to reported cases, are best known measures of transmissibility and transmission dynamics, and provide information for disease control programs [19]. For example, the herd immunity threshold and the critical level of vaccine coverage can be theoretically calculated from *R*_0_. Transmission rates of some childhood infections in Europe, North America and Africa temporally change [20–26]. The temporal change in a transmission rate can be described by the transmission rate seasonality, which is the amplitude of variation in the transmission rate. A small to medium level of seasonality in transmission rate can cause large amplitude fluctuations in the observed disease incidence [19]. By interacting with the number of susceptible and the transmission rate baseline, the transmission rate seasonality determines an annual or multi-annual cycle of observed incidence of these childhood infections [19, 24]. Because the disease transmission determines incidence dynamics, the transmission rate and its seasonality have been widely studied for childhood infections, such as measles, rubella, pertussis, mumps, and chickenpox, in Europe, America and Africa countries [20–26]. However, the transmission rate seasonalities in childhood infections in China are rarely investigated. Seasonality in transmission rate is an important but neglected consideration in the study of childhood infections in China. As to the HFMD transmission, there were only a few estimates about the average transmission rate and the basic reproduction number [1, 18, 27, 28], and one study showed a seasonal effective reproduction number for HFMD in Hong Kong [29]. However, the transmission rate seasonality for HFMD in mainland China has not been analyzed.

**Fig. 1:**
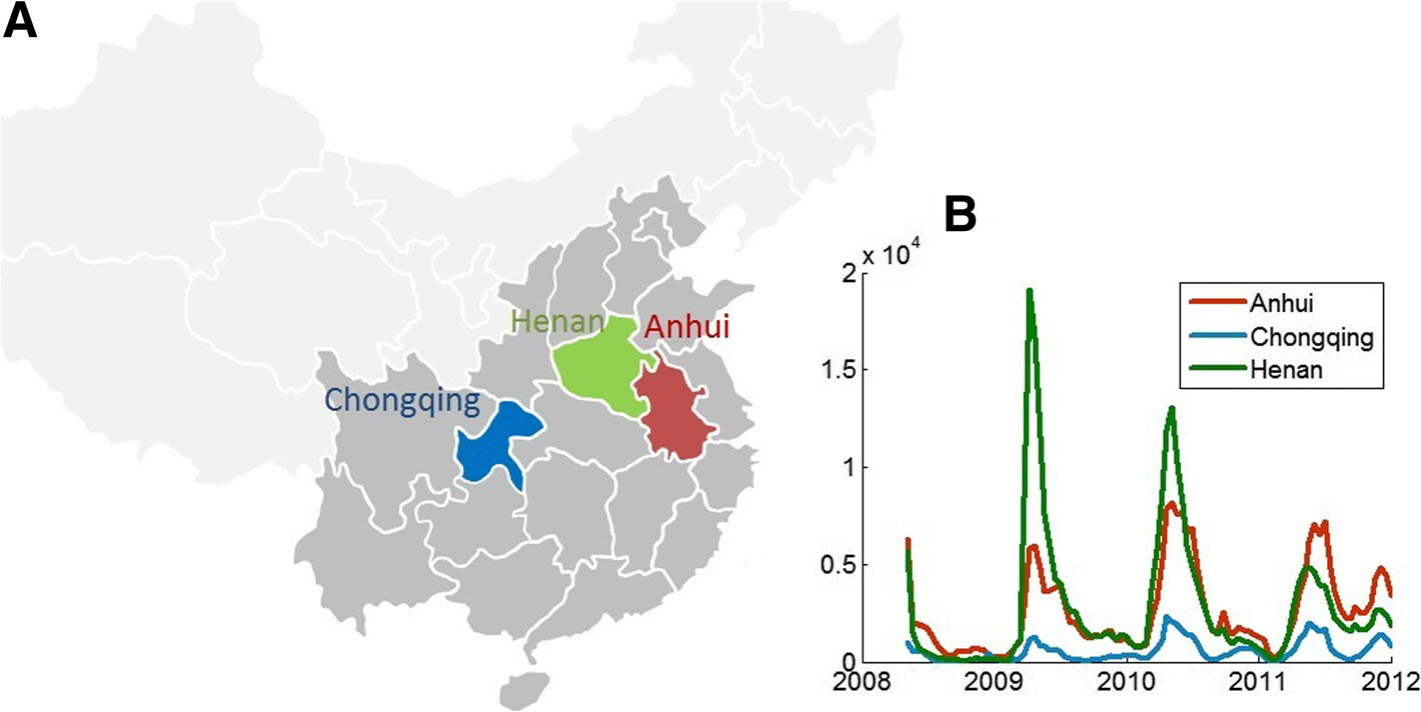
Locations and reported cases of Anhui, Henan and Chongqing in the map of mainland China. **a**) Locations of Anhui, Henan and Chongqing. Regions in dark grey are provinces that have severe HFMD outbreaks every year. **b**) Time series of reported HFMD cases in Anhui, Henan and Chongqing from May 2008 to December 2011

**Fig. 2:**
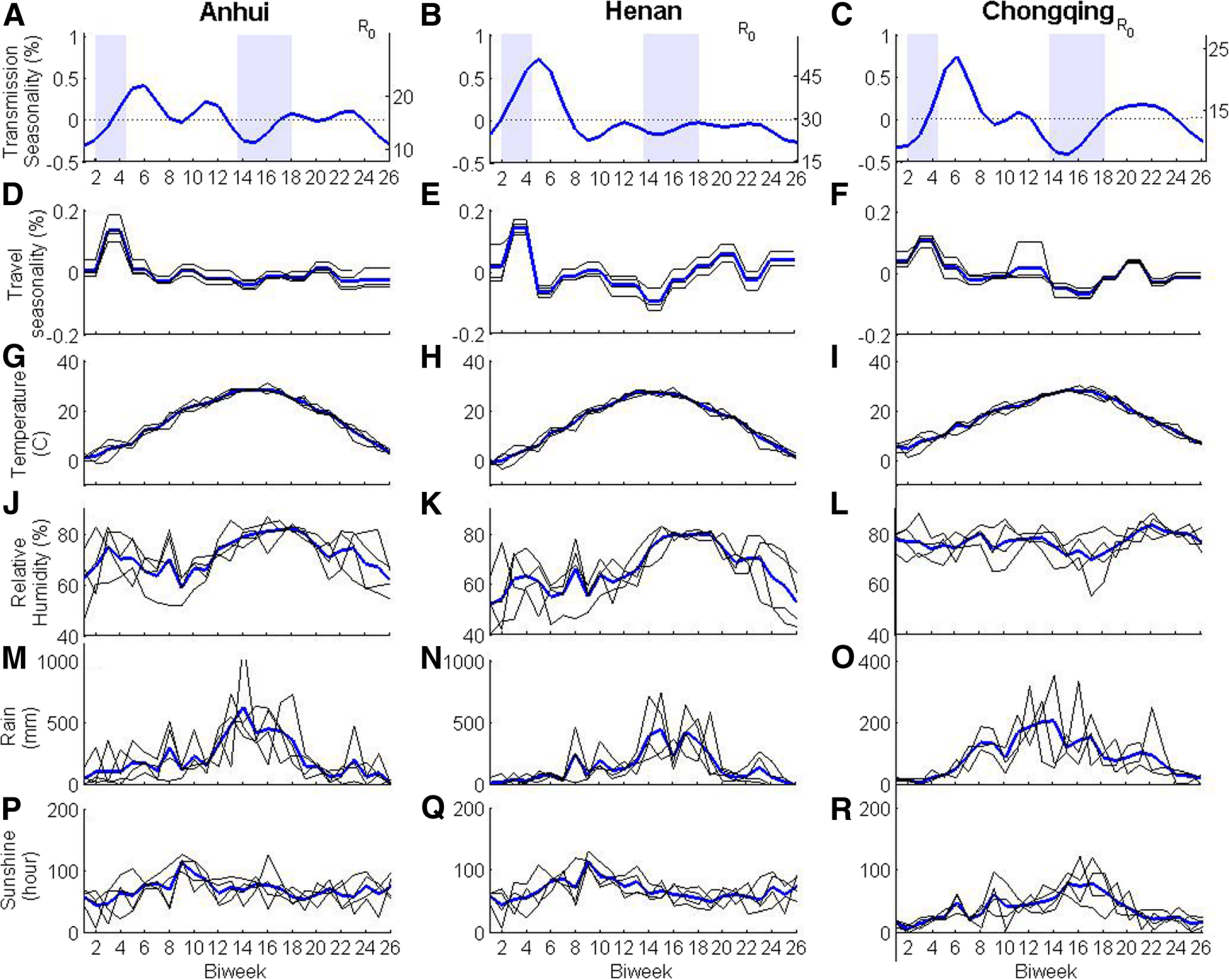
Transmission rates and potential influential factors for HFMD in Anhui, Henan and Chongqing. The MLE transmission rates of HFMD for **a**) Anhui, **b**) Henan and **c**) Chongqing. The band area of light blue corresponds to school vacations. *R*_0_ estimated for Anhui, Henan and Chongqing are consistent with other studies [1, 18, 27]. Blue lines in **d**), **e**) and **f**) are the averaged seasonality of highway passenger traffic from year 2010 to year 2013, and black lines are seasonality of highway passenger traffic for every year from year 2010 to year 2013. **g**) to **r**) time series of temperature, relative humidity, rainfall and sunshine hours in Anhui, Henan and Chongqing. Blue lines are the average of climatic factors from year 2008 to year 2011, and black lines are time series of climatic factors for every year from 2008 to 2011

In this paper, we will examine the transmission rate seasonality for HFMD in China, taking Henan province, Anhui province and Chongqing municipality as examples. We choose these three provinces because: 1) they have high population density, and locate in different parts (the north, east and west) of the southeastern region of China, in which severe HFMD outbreaks occurred [30] (Fig. 1a); 2) HFMD incidence cyclic patterns in Henan, Chongqing and Anhui represent respectively typical incidence patterns in northern and southern China and a mixture of northern and southern patterns (Fig. 1b). We are going to see whether the transmission rate seasonality of HFMD in three provinces are similar or different.

We will examine possible factors that cause the transmission rate seasonality of HFMD in the provinces. We will examine factors that have been studied for other childhood infections in developed countries and Africa countries, including the school terms and the seasonal migration of population. The temporally changed transmission rate can be described as *β*(*t*) = *c*(*t*) *p*, where *c*(*t*) is the temporally changed contact rate and *p* is the probability of being infected per contact. The seasonality in transmission rate is determined by the seasonal contact rate. It has been well documented that transmission rates of some childhood infections have annual seasonality, affected mainly by one dominant factor of human behavior, either the periodic children’s contact rate due to school terms in industrialized countries [17, 20–23], or the periodic population contact rate due to the seasonal migration or the seasonal population flux in developing countries [24–26]. However, we suspect that these two types of factors may work simultaneously on the transmission rate seasonality in HFMD in China.

The transmission seasonality of HFMD in China might be complicated. There should be many factors that can affect the HFMD transmission seasonality. Firstly, similar to that in the Western countries, school terms might have an impact on HFMD transmission rate seasonality because schools and kindergartens in China have winter and summer vacations that both are four to six weeks long, and the children density in kindergarten or school classrooms is higher than in industrialized countries. Secondly, China has a seasonal population flux every year, mainly the annual immigration forcing during Chinese Spring Festival at the end of January or on February [31]. The migration due to Chinese Spring Festival called the Spring Festival travel rush is the largest in the world, and lasts 40 days. MSN described that the Chinese Spring Festival travel rush was the “world’s largest annual human migration, a travel season 60 times larger than America’s worst Thanksgiving Day scramble” [32]. Thirdly, the population contact rate during the Chinese Spring Festival should be much increased due to the Chinese tradition of family gathering. During the Chinese Spring Festival, people return to their hometown to be with their families and to visit relatives and friends. Hence, the population contact rate in this period of time is highly increased. Fourthly, Meteorological factors may change the contact rate via changing human behavior such as gathering more indoor during winter or raining days [33]. For example, the measles transmission in Africa was affected by the raining season that caused the seasonal changing in the contact rate in urban [24]. Fifthly, viruses that cause HFMD transmit via multiple routes, including the fecal-oral route and the aerosol route, hence more factors will affect HFMD transmission than measles or rubella that are airborne transmitted infections. Most probably, HFMD in China has a complicated transmission seasonality, which was caused by combining effects of these above.

This paper is to analyze the transmission seasonality and possible determinants in HFMD in China. The transmission dynamics uncovered will provide information for further studies that can better understand HFMD transmission in China. Usually, for childhood infectious diseases, the transmission rate among children is different from that between children and adults, hence age-specific transmission rates are usually discussed. This paper is only a primary study for checking the overall seasonal pattern and its complexity of HFMD transmission rate, and a study of the possible influential factors. We will estimate HFMD transmission rate in the population without specifying age-specific contact rates. If transmission rate seasonality was discovered and any evidence indicated that school terms might have an important effect on HFMD transmission, age-specific transmission rate will be examined in our future work.

## Methods

Henan, Anhui and Chongqing are in different regions of China. Henan is in the middle part of China with the land area of 167,000 km^2^ and had the population of 94.0 million in the 6th national census of population in 2010. Anhui province is located in the eastern region of China and borders 6 provinces. It has the land area of 139,600 km^2^. In 2010, Anhui had a population of 59.5 million. Chongqing is located in southwest China and is the only municipality in the southwest. It has a land area of 82,400 km^2^, and had a population of 32.8 million in 2010. Both Anhui and Chongqing are in the subtropical climatic zone, and Henan is in the warm temperate zone.

### Data

In this paper, we used the reported HFMD cases and demographic data in Anhui, Henan and Chongqing to estimate seasonal transmission rates in the three provinces. To analyze possible factors for the transmission seasonality, we used meteorological data and traffic data of the provinces, and information about the time period of school opening and closure and Chinese Spring Festival. The data we used for our analysis are listed in the file Additional file 1.

The bi-weekly reported cases of HFMD in Anhui, Henan and Chongqing from the first week of 2008 to the 52nd week of 2011 were obtained from the Chinese Center for Disease Control and Prevention [34]. In the 17th weeks (the 1st week of May) of 2008, HFMD began to be put into the surveillance system in China. Hence the data we used spans May 2008 to December 2011.

The annual birth rates and the population of Anhui, Henan and Chongqing from 2008 to 2011 were obtained from the National Bureau of Statistics of China [35]. The average birth rate from 2008 to 2011 in Henan, Anhui and Chongqing were 11.49‰, 12.76‰ and 9.76‰ respectively, and they are consistent during the years we studied.

Biweekly meteorological data from 2008 to 2011, including mean temperature, relative humidity, precipitation and sunshine hours, were obtained from China Meteorological Data Service Center [36]. For the reason that we choose these meteorological factors, please refer [30].

Kindergartens in China usually have similar vacation times as schools. Because there is no official schedule for the kindergarten summer vacation, we assume that kindergartens and primary schools have the same schedule. The annual summer vacation is from the mid July to August 31st (weeks of 29–36). Usually, the winter vacation is from the middle of the 12th lunar month to the middle of the first lunar month (weeks 3 to 7 in 2009, weeks 6 to 10 in 2010, weeks 4 to 8 in 2011). We used February and the last 2 weeks of January as the winter vacation period.

The Chinese Spring Festival is a weeklong national holiday, marked according to the lunar calendar. However, population activities for the Chinese Spring Festival start from about one week before the Spring Festival Eve and ends after the 15th day of the first lunar month, the lantern’s day. During these three weeks, people spend a lot of time with family, gathering with friends and visit relatives. In this paper, we call the time period before and after the Chinese Spring Festival when contact rate is highly increased as the Chinese Spring Festival period. Considering the situation that the time window of the Chinese Spring Festival shifts every year because of the lunar calendar, we marked the Chinese Spring Festival period from mid January to the beginning of March.

The passenger traffic in China includes the highway passenger traffic, the air passenger traffic, the railway passenger traffic and the water passenger traffic. For provinces studied, we obtained the monthly highway passenger traffic from January 2010 from the Ministry of Transport of the People’s Republic of China [37], and the yearly total passenger traffic, yearly railway passenger traffic and yearly water passenger traffic from the National Bureau of Statistics of China [35]. The highway passenger traffic in Anhui or Chongqing accounts for more than 97% of the total passenger traffic in the province every year [35]. For the same time period, the highway passenger traffic in Henan accounts for more than 95.4% of the total passenger traffic [35]. The amount of highway passenger traffic can be considered to represent traffic patterns of the three provinces. The highway passenger traffic in Anhui, Henan and Chongqing increased gradually and steadily year by year. This increase was because of the improvement of road facilities and vehicle supplies, but not increased crowd on vehicles. In order to study the seasonal effect of the highway passenger traffic on the transmission rate of HFMD, we detrended the highway passenger traffic by removing the best straight-line fit from the data. We then calculated the traffic seasonality for each province by subtracting the detrended data with mean value and then dividing it by the mean.

The highway passenger traffic is for relatively shorter distance travel, because the average travel distance was around 66 km for Henan, 65 km for Anhui and 30 km for Chongqing respectively [37]. These travels should mostly happen within Henan, Anhui or Chongqing. According to our communication with the Ministry of Transport of the People’s Republic of China, the highway passenger traffic was counted based on the number of tickets of departure. For long distance travel in China, passengers usually take railway or airplane, which have average traveling distances of 400 km and 1500 km respectively. However, data of long and medium distance travels cannot be obtained and quantified. Since more than 95% of the total passenger travel was on the highway travel, and considering the availability of data, we do not analyze the passenger traffic of other traffic means assuming they have a similar seasonal pattern as the short distance travel.

### The stochastic model of HFMD transmission

Hand, foot and mouth disease transmission is a partially observed stochastic dynamical process. It has an underlying transmission process that state variables of susceptible and recovered individuals are not observed; and it also has an observation process that the infected individuals can be observed through reported cases. We model this partially observed stochastic dynamical process utilizing Partially Observed Markov Process (POMP) models [38, 39]. This stochastic model will be used to estimate the transmission rate seasonality of HFMD in selected provinces in China.

Our partially observed stochastic process model for HFMD has two parts: the transmission process model and the observation model.

#### The transmission process model

To describe the transmission process model, we first introduce its deterministic skeleton. The deterministic compartmental model that describes the HFMD transmission process is a SIR model. The population is divided into three compartments according to their infectious status: Susceptible (S), Infected (I) and Recovered (R). The number of susceptible, infected, recovered and the total population are denoted *S*(*t*), *I*(*t*), *R*(*t*) and *N*(*t*). The per capita rate at which susceptible individuals contract the infection is defined as the force of infection, λ(*t*). Then, the new infected are produced at the rate of λ(*t*)*S*(*t*).

The force of infection is modeled similar to [22],

1 $$ \lambda (t)=\left\{\beta (t)\frac{I(t)}{N(t)}+\psi \right\}\varepsilon (t) $$

The infected *I*(*t*) is one of the two sources of transmission to susceptible with transmission rate of *β*(*t*); the parameter *ψ* is the lower bound of the force of infection and can be seen as the transmission through viruses that are constantly carried in the population and in the environment; *ε*(*t*) is a process noise. The transmission rate was modeled using a B-spline such that the transmission rate can be estimated flexibly:

2 $$ \beta (t)=\exp \left\{\sum \limits_i^n{q}_i{\xi}_i(t)\right\}, $$

where *ξ*_*i*_(*t*) is a periodic B-spline basis with one year period, and *q*_*i*_ is the parameter that will be estimated. We used a B-spline with nine basis functions (*n* = 9) and degree of 3. The process noise *ε*(*t*) is assumed to be gamma distributed,

3 $$ \varepsilon (t)\sim \Gamma \left(1/\Theta, \Theta \right) $$

This gamma distribution has mean 1 and variance of Θ that will be estimated. The variance accounts for both environmental and demographic stochasticity.

The stochastic transmission process model is a bi-weekly discrete-time SIR model. In the stochastic discrete-time SIR model, the transition between compartments is a random process, in particular, the transition from susceptible to infected individuals follows a Poisson process with the parameter *λ*(*t*) that is described by eq. (1).

#### The observation model

In the observation model, we assume that bi-weekly cases reported in the model, *X*(*t*), are normally distributed:

4 $$ X(t)\sim N\left(\rho I(t),\tau I(t)\right) $$

where *ρ* is the reporting rate that will be estimated; the variance scales linearly with the mean, and the parameter *τ* will be estimated. Then cases reported in the model are rounded integer from *X*(*t*) if *X*(*t*) is positive, or are 0 otherwise.

In handbooks or fact sheets of HFMD on official websites of Health Department in different countries, the incubation period of HFMD is 3–7 days and the infected period is about 7–10 days [40–42]. A recent study support this incubation period that the estimated incubation periods were 4.4 (95% CI 3.8–5.1) days, 4.7 (95% CI 4.5–5.1) days and 5.7 (95% CI 4.6–7.0) days for children in kindergartens, primary schools and secondary schools respectively [43]. The serial interval in the model was assumed to be two weeks based on the fact that the incubation period of HFMD is 3–7 days and the infectious period is about 7–10 days.

We used the R package pomp to simulate the process and estimate parameters. The POMP version of the SIR model was fitted using data of bi-weekly reported cases of Anhui, Henan and Chongqing. Parameters were estimated by utilizing Maximization Likelihood by Iterated Particle Filtering (MIF), the function mif, in the R package pomp. Iterated filtering methods have been shown to solve successfully likelihood-based inference problems for epidemiological situations [39]. We used the IF2 algorithm of Ionides et al. (2015), which uses a random walk in parameter space to approach the Maximum Likelihood Estimate (MLE). At the end of time series, a new particle filter with a smaller random walk variance is selected. As the intensity of this random walk approaches zero, the modified model approaches the original model. We used 5000 particles and geometric cooling schedule.

Although age structure is usually taken into account for childhood infections, it is ignored for simplicity in this study. We want to first find whether there is any seasonal pattern in HFMD transmission rate, and to see the overall amplitude of seasonality of HFMD transmission.

### The regression model for effects of factors on transmission seasonality

To analyse the effects of factors on the transmission seasonality, we fitted a multiple regression model to the response, the transmission rate, with two categorical factors of the school terms and the Chinese Spring Festival period, and continuous variables of temperature, relative humidity, rainfall, hours of sunshine, and the highway passenger traffic. Because excessive correlations were present among the four meteorological factors, we first identify collinearity among factors by using variance inflation factors (VIF) and then remove individual factors that have high VIF values. A backward approach was used until all VIF values were below the threshold value of 10. After all factors with a higher VIF value were removed from the model, we used an analysis of variance, subtracting factors in a stepwise manner until the best fitting model was found. R, version 3.1.0 was used for the regression models.

For the categorical variable of school terms, we used three levels: summer vacation, winter vacation and school opening. Winter vacation of schools should be different from summer vacation because it coincides with the Chinese Spring Festival during which contact between children and adults may be increased. The categorical variable of the Chinese Spring Festival period has two levels, Spring Festival and none Spring Festival.

## Results

### Transmission rate seasonality

The MLE transmission rates of HFMD in Anhui, Henan and Chongqing are consistent and have complex seasonality (Fig. 2a-c), with two major seasonal waves: one higher peak in March and one smaller peak from September to the beginning of November. There is one minor peak in June. Lowest transmission rates happened in December, January and July. The MLE transmission rates increased dramatically from the end of January to the beginning of March, and then kept high during March, just after school resumed. This is the opposite of the expected correlation of transmission with warm weather for diseases transmitted through the fecal-oral route. The great increase in the transmission rate in February corresponded to the Chinese Spring Festival period, school winter vacation and the high level of highway passenger traffic in this month (Fig. 2d-f). The average temperature, relative humidity, rainfall and sunshine hours in Anhui, Henan and Chongqing had an annual seasonal pattern, but they were not consistent with the large increase in transmission rate in February (Fig. 2g-r).

The second major peak of seasonality in transmission from September to the beginning of November was during the fall semester of schools and kindergartens. The HFMD transmission rate in Henan from September to December was much lower than that in Anhui and Chongqing and below its average transmission rate. Many studies showed that the low transmission rate of childhood infectious diseases in several months after school started is attributable to susceptible exhaustion after the start of the school year [20, 44]. Hence, the decrease of HFMD transmission rate after peaks since April and November should be due to a similar reason, the depletion of susceptible.

Transmission rates after the Labor Day (May 1st or the 9th bi-week) had a brief rise. The increase of transmission rate from May to early June may be due to the nature of the pathogen that the transmission rate of this disease may be facilitated by warm weather or higher relative humidity [2, 4].

### Location effects

The complex seasonality in HFMD transmission rates for Anhui, Henan and Chongqing may be caused by several reasons. We used the multiple regression model to study effects of potential factors. The highway passenger traffic and the Chinese Spring Festival period seem to have delayed effects. Hence, we also fitted a regression model to the response with all the above parameters, however the highway passenger traffic and the Chinese Spring Festival period had different weeks of delay. *R*^*2*^s of models with four weeks delayed highway passenger traffic and four weeks delayed Chinese Spring Festival period were much higher compared with regression models with no delay, two-week delay and six-week delay. We also explored whether school terms also had any delayed effect, however, no delayed effect was found.

We first fitted a regression model with all variables that were introduced in the Method section and the location of provinces as a factor. Estimates of coefficients of the model with four weeks delayed highway passenger traffic and four weeks delayed Chinese Spring Festival period is listed in Additional file 2: Table S1. Henan had significantly higher HFMD transmission rate than Anhui, and even higher transmission rate during the Chinese Spring Festival period. Chongqing has a significantly lower transmission rate than Anhui. Since the location can explain the majority of the variance and transmission rates are significantly different between provinces, we analyze each province separately for the purpose of finding major causes of transmission rate seasonality.

### Combined effects of factors

The Chinese Spring Festival period, the highway passenger traffic, school terms and meteorological factors had combined effects on HFMD transmission in Anhui, Henan and Chongqing in China. The estimated coefficients of minimal adequate models for Anhui, Henan and Chongqing are listed in Table 1. The Chinese Spring Festival period and school terms had significant effects on the HFMD transmission rate seasonality in all three provinces.

**Table 1:** Minimal adequate models for Anhui, Henan and Chongqing

	Anhui		Henan		Chongqing	
Variable	Coefficients [90%CI]	t value	Coefficients [90%CI]	t value	Coefficients [90%CI]	t value
Intercept	− 182.68 [− 367.70,11.33]	−1.54	254.03* [61.49,446.58]	2.16	348.50*** [327.49,369.51]	27.20
School summer vacation	−89.79*** [− 124.99,-54.59]	−4.18	−15.22 [− 88.17,57.73]	−0.34	− 107.91** [− 155.54,-60.28]	−3.72
School winter vacation	9.63 [−25.86,45.14]	0.45	183.90*** [118.9248.88]	4.64	− 104.61** [− 158.37,-50.85]	−3.19
Spring Festival (4 wks delay)	100.7*** [59.22,142.18]	3.98	394.87*** [331.8457.93]	10.27	191.72*** [143.88,239.56]	6.57
Highway passenger traffic (4 wks delay)	9.48*** [5.91,13.05]	4.35	10.58*** [6.16,15.01]	3.91	–	–
Relative Humidity (%)	6.30*** [4.17,8.43]	4.85	6.38** [3.43,9.33]	3.55	–	–
Temperature (°C)	–	–	–	–	–	–
Sunshine (hours)	1.95** [1.03,2.86]	3.50	–	–	–	–
Rainfall (mm)	–	–	–	–	–	–
Adjusted Multiple *R*^2^	0.88		0.89		0.72	
F-statistic	30.66		43.69		22.86	
Degree of freedom	19		20		22	
p-value	8.54e-9		4.34e-10		5.91e-7	

*** *p*-value < 0.001, **p-value< 0.01, * *p*-value < 0.05, .*p*-value < 0.1

Cells with “-” indicate that the variables or factors were not included in the minimal adequate model

Among the three provinces, Chongqing has the simplest combined effects of factors on the HFMD transmission rate. The minimal adequate model for Chongqing has adjusted R^2^ of 0.72 and showed significant effects of the Chinese Spring Festival period and school terms on HFMD transmission rate (columns 6 and 7 in Table 1). The Chinese Spring Festival period and school terms together explained the majority of variance in HFMD transmission rate in Chongqing. Highway passenger traffic and meteorological factors did not have a significant effect on the HFMD transmission rate. The HFMD transmission rate in Chongqing was significantly decreased during school winter and summer vacations. Since the time periods of the winter vacation and the Chinese Spring Festival period had much overlap and school winter vacation had a negative effect, the large increase in transmission rate during February should be caused by the effect of the Chinese Spring Festival period. The Chinese Spring Festival period explained the large variance in transmission rate happened during February, and its effect diminished after March. The second transmission peak in Chongqing should be dominantly caused by school terms.

For Anhui province, the four types of factors had combined effects. Meteorological factors of relative humidity and sunshine hours, the Chinese Spring Festival period, the highway passenger traffic and school terms had significant effects on the HFMD transmission rate in Anhui (columns 2 and 3 in Table 1). To see the relative importance of the four types of drivers, we fitted data to single variable models that consider only one type of variable, including the meteorological factors model, the Chinese Spring Festival period model, the highway passenger traffic model, and the school terms model. The only Chinese Spring Festival period model or the only highway passenger traffic model showed significant effects of the factors on the HFMD transmission rate, with the model adjusted *R*^2^ of 0.61 and 0.69 respectively (Additional file 2: Table S2). The seasonality of the highway passenger traffic mainly happens during the Chinese Spring Festival period. When the model includes both the highway passenger traffic and the Chinese Spring Festival period, the model power of adjusted *R*^2^ increased to 0.75. The highway passenger traffic and Chinese Spring Festival period together explained the majority of variance of the transmission rate. In single variable models, meteorological factors or school terms did not show significant effects on the HFMD transmission rate in Anhui, however, they were included in the minimal adequate model, and increased the adjusted *R*^2^ of minimal adequate model to 0.88. In the minimal adequate model, the school summer vacation gave a significantly lower transmission rate than during school opening; the winter vacation did not have significant difference than school opening.

HFMD transmission in Henan was quite similar to that in Anhui. The minimal adequate model showed that the Chinese Spring Festival period, school terms, meteorological factors of relative humidity and the highway passenger traffic had significant effects on the HFMD transmission rate in Henan. The Chinese Spring Festival period and the highway passenger traffic explained the majority of variance in transmission rate in Henan (Additional file 2: Table S3). Different from Anhui, the HFMD transmission rate in Henan significantly increased during school winter vacation.

The HFMD transmission rate increased dramatically in February and peaked at the beginning of March in all three provinces. This large variance in transmission rate during February and March did not agree with temporal changes of any meteorological factor. Meteorological factors may have effect on the transmission rate as shown in minimal adequate models in Anhui and Henan in Table 1, however, the Chinese Spring Festival period and the highway passenger traffic explained the majority of the variance. Meteorological factors may work as confounders.

For other infections such as influenza, absolute humidity rather than relative humidity have been found to be more closely related to transmission [45]. We included the absolute humidity in the regression model to replace the relative humidity and temperature. Results showed no significant effect in absolute humidity on the HFMD transmission rate in all three provinces.

## Discussion

This paper studied the seasonality of HFMD transmission rate and its possible influential factors in China, taking Anhui, Henan and Chongqing as examples. The transmission rate of HFMD for all three provinces had a complex seasonality pattern with one major peak after February, and a smaller peak with longer duration from September to November. The dramatic increase in HFMD transmission rate in February coincides with the time period of Chinese Spring Festival, school winter vacation and annual peak of highway passenger traffic. High transmission rates from September to the beginning of November in Anhui and Chongqing coincide with school opening.

The major variance in transmission rate happened during February and March. The Chinese Spring Festival period and the short-distance population flux (the highway passenger traffic) appear to be the dominant factors for the seasonality in transmission rate in the first few months of the year. In the other months of the year, effects on transmission rate were dominated by school terms, the highway passenger traffic and meteorological factors in Anhui. Transmission rates of some childhood infections in Europe, America and Africa countries also have complex transmission seasonality with multiple peaks [23, 25, 46]. Dominant factors for some of the complex seasonal transmission were not studied, and for some others was either school terms or population flux. Unlike these childhood infections in other countries that have one dominant factor for transmission seasonality, different seasonal factors dominated the effect on the HFMD transmission rate seasonality in different period time of the year in China.

The dominant factor for transmission seasonality of a childhood infectious disease may depend on the transmission level of the disease. A disease with a higher transmission level mainly infects younger children [47]. For example, in Niger, the average age of measles infection is 2 years because of high transmission rate [24]. Hence, the majority of infected children did not go to school. Seasonality in measles transmission in Niger is more affected by seasonal contact rate of the whole population. While in industrialized countries, measles had relatively lower transmission level than in Africa, and the average age of measles infection in the pre-vaccination era was 4–6 years [47], hence transmission seasonality was more affected by the seasonality in children’s contact. China has a situation that children older than 3 years go to kindergartens and stay in kindergartens even longer per day than students in elementary schools. The average age of HFMD infection in China is 2.7 years. Most infected children are 5 years old and younger [1]. HFMD transmission in 2 years and younger should be affected by contact rate in the population, and HFMD transmission in 3 to 5 years old should be more affected by children’s contact. This is why the transmission rate seasonality of HFMD in China is complex. The contact rate seasonality in both children and population should affect the HFMD transmission rate in China. During February and March, population contact rate varied much greater than children contact rate, hence factors that caused population contact rate changing could dominant effects on HFMD transmission rate seasonality during this period of time. The second major seasonality of HFMD transmission rate in Chongqing was caused by the increased contact rate among children during school opening. The transmission rate seasonality of measles in Europe in the pre-vaccination era is about 5–20% [19]. If we consider months from April to December when the Chinese Spring Festival period was not in effect, transmission rate seasonality of HFMD during this period of time is 25% in Chongqing and 9% in Anhui, which are kind of similar to transmission rate seasonality of measles in Europe in the pre-vaccination era.

The transmission rate seasonality of measles in Africa is very consistent with the seasonal nature of the rainy season [24]. The effect of the raining season was indirect, because the raining season caused the seasonality of population density in urban, which affect the transmission rate of measles. Unlike raining season in Africa, there is no meteorological factor that can cause the high seasonality in transmission rate in February in Anhui, Henan and Chongqing in China. Meteorological factors might have an effect on the baseline transmission [31], but they cannot solely explain the transmission seasonality and they only have minor effects on transmission rate seasonality.

The peak time of incidence cycle often lags behind the peak time of transmission rate, because it takes time from the beginning of the infection to the outbreak. Based on the intensity of the transmission of the disease, the peak time of the transmission rate of a childhood infection is one to three months earlier than the peak time of incidence, for example, polio in the United States [22] and measles in Europe [19]. The results of this study show a similar situation: the high transmission rate in March lead to the large peak of incidence during May or June, and the higher transmission rate from September to October lead to the peak of incidence during November. It should be noticed that, incidence seasonality and transmission rate seasonality are different concepts. Incidence seasonality or incidence cyclic patterns of childhood infectious diseases may be annual or multi-annual, while transmission rate seasonality of these diseases is annual. HFMD incidence shows annual or multi-annual cyclic patterns in Japan, Singapore, Malaysia and other countries [4, 7, 8]. In China, HFMD incidence has obvious annual cyclic patterns [1, 9]. Reasons for cyclic patterns of HFMD incidence remain unclear, but were mainly studied for meteorological factors by associating meteorological factors with reported cases [2, 4, 48, 49]. However, meteorological factors have inconsistent and even conflicting effects in different regions in China [30]. Meteorological factors are clear driving factors for the cyclic pattern of vector-borne diseases, because they can seasonally influence vector abundance [50]. However, for childhood infections such as measles, rubella, pertussis, mumps in Europe, America countries and Africa countries, meteorological factors did not cause the cyclic pattern of incidence. As discussed in the Introduction session, the cyclic nature of incidence is determined by transmission rate seasonality, and whether the cyclic pattern of incidence is annual or multi-annual should be determined by transmission rate baseline, transmission rate seasonality and the number of susceptible. Here we showed that meteorological factors cannot solely explain transmission rate seasonality in HFMD, hence it is impossible for it to solely explain cyclic pattern in HFMD. In this paper we analyzed the transmission rate seasonality and its influential factors for HFMD, next we will investigate how seasonality in transmission, baseline transmission and birth rates interact to generate annual, bi-annual or multi-annual cyclic pattern of HFMD incidence in different countries in Asia and Pacific regions.

We did not use an age-structured model, hence we did not separate contact rates in children and in adults. The transmission rate and its seasonality could be estimated more precisely if contact rate in ages were considered. Besides the lack of age-specific transmission rates in the model, this paper used regression models rather than dynamic models to identify the influential factors for the transmission rate seasonality. Using a regression model is an informative first step to identify key influential factors from several different types of possible factors. In our future work, we are going to build in age structure and seasonal drivers into stochastic transmission models to fit observed incidence data.

The Chinese Spring Festival is usually a week holiday in February, during which the population return home and back to the countryside, resulting in increased passenger traffic. Anhui is top among provinces for positive migration during the Spring Festival. Its immigration and emigration are the highest in February. Hence, in February in Anhui, the seasonality in total population flux should be much higher than the seasonality in with-in province population flux. Henan and Chongqing have the similar situation. Using highway passenger traffic only underestimated the seasonality of population migration during Spring Festival and hence underestimated the effect of population flux. If the time series of total passenger traffic can be considered in the model, the effect of seasonal population flux on the seasonality in HFMD transmission should be even higher. Future work should focus on the collection of migration data among regions and the effect of migration on the persistence of HFMD in the country.

## Conclusion

The transmission rate of HFMD in three provinces in mainland China had complex seasonality. The Chinese Spring Festival, the population flux and (or) school terms explained the majority of the transmission rate seasonality of HFMD, and they drove HFMD transmission rate seasonality in different time periods of the year. The Chinese Spring Festival dominantly caused the dramatic increase of the HFMD transmission rate during February.

## Additional files

Additional file 1: RegressionData. The data used for the regression model. Data in this file include the meteorological data, the highway passenger traffic data, whether schools are in vacations or opening, and information of the Chinese Spring Festival period. (CSV 6 kb)

Additional file 2: **Table S1.** Regression models for transmission rates in Henan, Anhui and Chongqing. **Table S2**. Model coefficients of single variable models, model of traffic and Spring Festival, and minimal adequate model for Anhui Province. **Table S3**. Model coefficients of single variable models, model of traffic plus Spring Festival, model of traffic plus Spring Festival and school terms, and minimum adequate model for Henan province. (DOCX 19 kb)

Additional file 3: The number of births and population in Anhui, Henan and Chongqing from 2008 to 2011. (XLSX 8 kb)

## Data Availability

Data analyzed during this study, such as meteorological data and population flux data, school vacations and openings, and the Chinese Spring Festival, are included in this published article and its Additional file 1. Data of HFMD reported cases for Chongqing, Anhui and Henan in China were obtained from The Data-center of China Public Health Science via communication. A liability statement was made by authors of this paper to The Data-center of China Public Health Science for not releasing the data to public. However monthly reported cases data can be downloaded from the website of The Data-center of China Public Health Science. Our analyzed data supporting the conclusions of this article are included within the Additional files 1, 2 and 3.

## Abbreviations

CA16: Coxsackievirus A16
EV71: Enterovirus 71
HFMD: Hand, Foot and Mouth Disease
MIF: Iterated Particle Filtering
MLE: Maximum Likelihood Estimation
POMP: Partially Observed Markov Process
VIF: Variance Inflation Factors
