# Supplementary material for: The complex transmission seasonality of hand, foot, and mouth disease and its driving factors
Source: BMC Infect Dis. 2019 Jun 13;19:521. doi: 10.1186/s12879-019-4153-6 (PMC6567494; doi:10.1186/s12879-019-4153-6)
Supplement: Supplementary file 2 — Table S1. Regression models for transmission rates in Henan, Anhui and Chongqing. Table S2. Model coefficients of single variable models, model of traffic and Spring Festival, and minimal adequate model for Anhui Province. Table S3. Model coefficients of single variable models, model of traffic plus Spring Festival, model of traffic plus Spring Festival and school terms, and minimum adequate model for Henan province. (DOCX 19 kb) [file 12879_2019_4153_MOESM2_ESM.docx]

1. Results of regression model considering sites

Considering the situation that residuals of multiple linear regression model without interaction decreases with fitted values, interaction factor were included in the minimal adequate model. The interaction of the Spring Festival period with one month delay and the highway passenger traffic with one month delay has positive effect on the transmission rate. The only location model has adjusted R^2^ of 0.64.

Table S1. Regression models for transmission rates in Henan, Anhui and Chongqing

| Variable | Regression coefficient | *t* value | *p*-value |
| --- | --- | --- | --- |
| Intercept | 157.078 . | 1.786 | 0.078 |
| Spring Festival  (4 wks delay) | 92.178* | 2.190 | 0.032 |
| Highway passenger traffic  (4 wks delay) | 4.42 . | 1.68 | 0.097 |
| Chongqing | -73.833*** | -3.538 | 0.0007 |
| Henan | 320.766*** | 15.227 | < 2e-16 |
| School opening | - | - | - |
| Relative Humidity (%) | 3.145* | 2.591 | 0.0116 |
| Temperature (°C) | - | - | - |
| Sunshine (hours) | - | - | - |
| Rainfall (mm) | - | - | - |
| Traffic:Spring Festival | 7.96* | 2.026 | 0.047 |
| Spring Festival:Chongqing | 21.413 | 0.418 | 0.677 |
| Spring Festival:Henan | 304.766*** | 5.933 | 1e-7 |
| Adjusted Multiple *R*^2^ | 0.91 | | |
| F-statistic | 99.4 | | |
| Degree of freedom | 69 | | |
| p-value | <2.2e-16 | | |

*** p-value <0.001, **p-value< 0.01, * p-value <0.05, .p-value <0.1

Cells with “-“ indicate that the variables or factors were not included in the minimal adequate model.

1. Results of regression models for three provinces

For all three provinces, we found there is no significant interaction effects between the Spring Festival and Travel.

Table S2. Model coefficients of single variable models, model of passenger traffic and Spring Festival, and minimal adequate model for Anhui province

|  | Only Spring Festival | Only traffic | Passenger traffic + Spring Festival | Minimal adequate model |
| --- | --- | --- | --- | --- |
| Intercept | 377.40*** | 406.699*** | 393.150*** | -182.6846 |
| Spring Festival period | 170.84*** |  | 81.584* | 100.7060*** |
| Highway passenger traffic |  | 15.548*** | 10.405*** | 9. 4826*** |
| Winter vacation |  |  |  | 9.6348 |
| Summer vacation |  |  |  | - 89.7892*** |
| Relative Humidity |  |  |  | 6.2987*** |
| Sunshine hours |  |  |  | 1.9477** |
| Adjusted R^2^ | 0.6128 | 0.6959 | 0.7542 | 0.8768 |
| F-statistic | 40.56 | 58.22 | 39.36 | 30.66 |
| Degree of freedom | 24 | 24 | 23 | 19 |
| p-value | 1.386e-06 | 7.223e-08 | 3.758e-08 | 8.549e-09 |

*** p-value <0.001, **p-value< 0.01, * p-value <0.05, .p-value <0.1

School terms and meteorological factors do not show significant effects in their single variable models.

Table S3. Model coefficients of single variable models, model of traffic plus Spring Festival, model of traffic plus Spring Festival and school terms, and minimum adequate model for Henan province

|  | Only Spring Festival | Only Passenger traffic | Only School terms | Passenger traffic+  Spring  Festival period | Passenger traffic +  Spring Festival period + School terms | Minimal adequate model |
| --- | --- | --- | --- | --- | --- | --- |
| Intercept | 683.15*** | 751.27 *** | 736.06*** | 691.11*** | 667.07*** | 254.033 *** |
| Spring Festival | 449.30*** |  |  | 394.77** | 371.40*** | 394.868 *** |
| Passenger traffic |  | 18.45** |  | 7.9305* | 904.41* | 10.586 *** |
| Winter vacation |  |  | 239.80* |  | 163.50** | 183.897*** |
| Summer vacation |  |  | - |  | - | - |
| Relative Humidity |  |  |  |  |  | 6.384 ** |
| Sunshine hours |  |  |  |  |  |  |
| Adjusted R2 | 0.7267 | 0.2927 | 0.1335 | 0.7675 | 0.8373 | 0.8952 |
| F-statistic | 67.49 | 11.34 | 2.925 | 42.26 | 33.16 | 43.69 |
| Degree of freedom | 24 | 24 | 23 | 23 | 21 | 20 |
| p-value | 1.966e-08 | 0.002549 | 0.07381 | 1.984e-08 | 8.466e-09 | 4.34e-10 |

*** p-value <0.001, **p-value< 0.01, * p-value <0.05, .p-value <0.1

Meteorological factors do not show significant effects in the single variable model.
